# Generator Maintenance Scheduling using Exchange Market Algorithm

**DOI:** 10.1016/j.mex.2020.100932

**Published:** 2020-05-23

**Authors:** Learnmore Moyo, Nnamdi I. Nwulu, Uduakobong E. Ekpenyong

**Affiliations:** a University of Johannesburg; b Aurecon Group PTY Ltd

**Keywords:** Constraint Handling, Metaheuristic algorithm, Generator maintenance, Constraint violation, Maintenance window

## Abstract

The Generator Maintenance Schedule model is formulated mathematically as a highly constrained combinatorial optimization problem and it is obligatory to implement a suitable optimization tool to determine the best feasible maintenance schedule. The maintenance schedule obtained has to meet a number of power system constraints. There is increased research in the development of approximate solution methodologies such as heuristic and meta-heuristic techniques [1]. Unlike mathematical methods, metaheuristics can obtain an optimal solution to a complex problem fast and are not subjected to limitations such as linearity, continuity, differentiability and convexity that are faced by mathematical programs [2]. This work presents the application of Exchange Market Algorithm (EMA) to find an optimal maintenance schedule. The algorithm is customized to achieve the following:

• Selecting the initial population within the maintenance window constraint to enable faster convergence.
• Adapt the algorithm to give discrete solutions.
• Penalty function included for constraint handling.

## Graphical abstract

**Figure:**
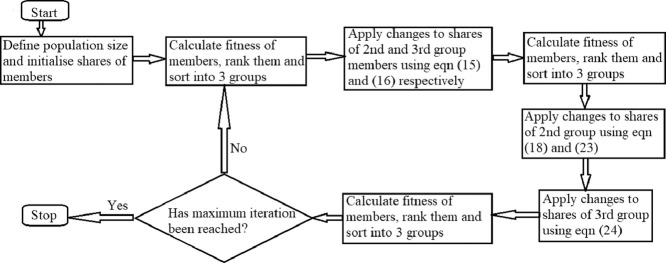

Specifications table

**Table:** 

Subject Area	• Energy
More specific subject area:	*Generator maintenance scheduling using a metaheuristic algorithm, EMA, ensuring that maintenance schedule meets all the power system constraints.*
Method name:	*Exchange Market Algorithm*
Name and reference of original method	*N. Ghorbani and E. Babaei, "Exchange Market Algorithm," Applied Soft Computing, vol. 19, pp. 177-187, 2014.*
Resource availability	*Matlab, GNU Octave*
*Type of Submission*	*Direct Submission*

## Method details

Metaheuristics have become popular as optimization techniques as they are able to overcome the shortfalls of exact solution methods such as long execution times for large problems which are often encountered in real life. Metaheuristic algorithms can provide reliable solutions within a short time although the solutions may not necessarily be optimal. They are nature inspired, for example Particle Swarm Optimization (PSO) is inspired by the ability of flocks of birds, schools of fish and herds of animals to adapt to their environment. A number of meta-heuristic algorithms have been developed over the years. In [3] a robust particle swarm optimization (PSO) algorithm is used to optimize reliability and economic cost objective functions. Genetic Algorithm (GA) is used in [4] for reliability objective function and in [5] for an economic cost objective function. Differential evolution is used in [6] to optimize a GMS problem. The other common metaheuristic method that is used is Simulated Annealing (SA) [7]. Metaheuristic algorithms are also used in combination to give hybrid metaheuristics, which improve the convergence of the algorithms. In [7], a GA/SA hybrid approach is also used the study concluded that hybrid approaches are less sensitive to variations of technique parameters.

One of the most recently developed metaheuristic algorithms is the Exchange Market Algorithm, EMA [8]. In the same fashion as other metaheuristic algorithms like PSO [9], it is population based. It is suitable for solving continuous non-liner optimization problems which are so common. The algorithm is inspired by the trading of shares on the stock market. In a stock market, each broker buys and sells shares, taking a certain level of risk, in order to increase their share portfolio. In EMA, each individual in the exchange market is a solution to the problem. The individuals compete to have the best share value and be ranked at the top. There are two different modes of the exchange market algorithm which emulate how certain conditions in real world affect the stock market. The first mode is the balanced or non-oscillating mode. In the balanced mode, individuals in the lower ranks try to use the experience of the high ranked shareholders in order to improve their share value and be recruited in to the elite class. The second mode is the oscillating mode in which the market is unstable and individuals tend to take calculated risks by identifying other shares that can improve their overall ranking.

All metaheuristic algorithms use a search operator and an absorbing operator to find the optimal value of a problem. These operators which are usually nature-inspired are used to generate and organize random numbers. In EMA, the generation and organizing of random numbers is done in an effective way because of the use of not one, but two search and two absorbent operators. This leads to an increased chance of locating the global optimum.

Metaheuristic algorithms are faced with issues of trapping in local optimum points leading to early convergence, inability to find adjacent points of the optimum and convergence to non-similar solutions [8]. Because EMA uses two efficient search and absorbing operators, it can overcome the limitations that are faced by other algorithms.

In each market mode of EMA, the individuals buy and sell their shares (the variables of the problem). At the end of the trading cycle (iteration), the fitness of each individual is evaluated and they are sorted according to their share values. After sorting according to their fitness, the individuals are then divided into three groups, first, second and third.

The first group is made up of the elite members who have the best solutions. They are generally comfortable with their share portfolio and remain unchanged.

The members of the second and third groups aim to be upgraded into the first group. They trade their shares using different equations with the hope of ending up with a better fitness. In a balanced market mode, the individuals in the second and third groups select shares which are the same or similar to those of the members of the first group. In this mode, the algorithm seeks to promote members of the lower groups to the first group.

In the oscillating mode, the individuals in second and third group take high risks in their trading because they have low fitness. They seek new unknown points and thus widen the search space.

## Application of EMA to GMS

The MATLAB code of EMA as implemented on 12 different benchmark functions is available online on the following link:

https://www.researchgate.net/publication/301613121_software_code_of_Exchange_Market_Algorithm_EMA_in_Matlab_for_solving_optimization_problems

There are four function files namely, *EMA, fitness, Initial, notoscillation* and *oscillation.* The initialization of the variables and parameters used in the algorithm is done in the function *Initial*.

For the GMS problem, the solution is a vector of maintenance start times. The total number of generating units in this instance is 157 and therefore the parameter num_par which represents the number of variables of the problem is 157. The other parameters that are initialized are the population size, number of iterations, percentage of members in the first, second and third groups for oscillating and non-oscillating mode and the parameters that determine how the algorithm searches the solution space.

The operating principle of the algorithm as applied to the GMS problem is detailed in the following steps.

1. In the main function, *EMA*, initialize the solution vector, pop, for all members of the population with maintenance start times that are fall within the earliest and latest maintenance start period of each generating unit. This is accomplished by the following code:

%**************************************************************************************

Num_Gen = 157;      % number of generators

Num_Periods = 365;  % number of time periods

limit_pop_up=ones(num_pop,num_par); % matrix of latest start times

limit_pop_dn=ones(num_pop,num_par); % matrix of earliest start times

pop = zeros(num_pop,num_par);         % matrix of population

global iteration;

%% first iteration

iteration=1;

for a = 1:num_pop

    for b = 1:Num_Gen

        up = Latest(b);

        dn = Earliest(b);      

       

        limit_pop_up(a,b)=limit_pop_up(a,b)*up;

        limit_pop_dn(a,b)=limit_pop_dn(a,b)*dn;

       

        pop(a,b) = round(dn+rand*(up-dn));

    end           

end

%************************************************************************************

2. The fitness of each member of the population is then determined by calculating the value of the objective function. The function *Fitness* does the evaluation. The code was amended in order to be suitable to evaluate the objective function of the GMS problem. A 157 × 365 (number of generators by number of time periods) binary matrix is created that has values of 1 when a generator is on maintenance and 0 otherwise. Another matrix showing the running status of generators is also created.

%************************************************************************************

for k=1:num_pop

    Maint_Start = ([pop(k,1:Num_Gen)]);      %Maintenance Start time vector

    On_Maintenance = zeros(Num_Gen,Num_Periods); %Maintenance schedule        initialisation

   %create binary matrix showing generator on maintenance 1 else 0

    for j=1:Num_Gen

        for l=Maint_Start(j):Maint_Start(j)+MaintenanceDuration(j)-1

            On_Maintenance(j,l)=1;           

        end

    end

    %create binary matrix showing running generators   

    for u=1:Num_Gen

        if(Gmax(u)>0)

            for v=1:Num_Periods

                Running(u,v)=not(On_Maintenance(u,v));

            end

        end

    end

%************************************************************************************

## Constraint Handling

Metaheuristic Algorithms are generally suited for unconstrained optimization problems. The handling of constraints is achieved by adding penalty functions to the objective function which transforms the constrained optimization problem to an unconstrained one [5,9]. The penalty function penalizes infeasible solutions by reducing their fitness based on the extent of constraint violation thereby pushing non-feasible solutions out of the high ranked solutions.

For this study, there are four constraints, maintenance window, maintenance duration, capacity plus minimum reserve satisfaction and exclusion constraint which does not allow some generators to go on maintenance at the same time. The first two are implemented in the generation of maintenance start times and in building the matrix that shows generators on maintenance respectively. The penalty function method is applied to the exclusion constraint and the capacity plus minimum reserve constraint.

The equation for the capacity constraint is given below:

(1) $$\sum_{i\varepsilon I}Gmax_{i,t}-\sum_{i\varepsilon I}\sum_{t\varepsilon T}Gmax_{i,t}.x_{i,t}\geq \;D_{t}+R_{t}$$

Where *Gmax*_*i, t*_ is maximum capacity of generator *i, x*_*i, t*_ a binary variable indicating whether a generator is on maintenance, *D_t_* is the demand at time *t* and *R_t_* is the minimum reserve. The penalty function, *p*_1_(*x*), for an inequality is given as:

(2) $$p_{1}\left(x\right)=\text{max}{\left\{g_{j}\left(x\right),0\right\}}^{2}$$

This is implemented in code as follows:

%************************************************************************************

%Calculation of capacity violation

    CapacityViolation = max(0,(Demand+2000)-AvailableCapacity);

    CV =500*iteration*sum(CapacityViolation.^2);

%************************************************************************************

The capacity violation is multiplied by a constant, 500, and a variable, iteration, which denotes the iteration number so that as the simulation proceeds, the fitness of infeasible solutions is greatly reduced.

For the exclusion constraint, the units that are not supposed to go on maintenance at the same time are put into exclusion sets. The exclusion constraint violation is computed in the same manner as above.

%************************************************************************************

%Exclusion of same generator units and violation

    ES1 = [On_Maintenance(1:2,:)];

    ES2 = [On_Maintenance(5:6,:)];

    ES3 = [On_Maintenance(9:10,:)];

    ES4 = [On_Maintenance(60:61,:)];   

    ES5 = [On_Maintenance(133:134,:)];

    ES6 = [On_Maintenance(136:137,:)];

    ES1v = max(0,sum(ES1,1)-1);

    ES2v = max(0,sum(ES2,1)-1);

    ES3v = max(0,sum(ES3,1)-1);

    ES4v = max(0,sum(ES4,1)-1);

    ES5v = max(0,sum(ES5,1)-1);

    ES6v = max(0,sum(ES6,1)-1);

   

    %Calculation of Exclusion Violation

EV=500*iteration*(sum(ES1v.^2)+sum(ES2v.^2)+sum(ES3v.^2)+sum(ES4v.^2)

+sum(ES5v.^2)+sum(ES6v.^2));

%************************************************************************************

The penalty functions are then added to the objective function to give a non-constrained problem and the fitness evaluated. The members of the population are then ranked and classified into the three groups according to fitness.

3. Variations are then applied to the shares (maintenance start times) of the members of the second and third group using the function *nooscillation* which indicates normal market mode. The function code, which is originally for continuous variables, was edited so that it can give the integer values of maintenance start time. The was achieved by introducing the matlab function *ceil*. The edited code for *notoscillation* is shown below.

%************************************************************************************

function pop=notoscillation(pop)

[num_pop,num_par,num_iter,num_pop11,num_pop12,num_pop13,num_pop21,

num_pop22,num_pop23,g1,g2]=Initial;

for j=num_pop11+1:num_pop

   if j<=num_pop11+num_pop12

       person(1)=ceil(num_pop11*rand);person(2)=ceil(num_pop11*rand);

r1=rand;

        pop(j,:)=round(r1*pop(person(1),:)+(1-r1)*pop(person(2),:));

%eq 14 in article

   else

       c1=2;c2=2;r1=rand(1,num_par);r2=rand(1,num_par);

       person(1)=ceil(num_pop11*rand);person(2)=ceil(num_pop11*rand);

       comp(j,:)=c1*r1.*(pop(person(1),:)-pop(j,:))+c2*r2.

*(pop(person(2),:)- pop(j,:));%

       pop(j,:)=round(pop(j,:)+0.8*comp(j,:));% eq. (15)in article

   end

end

end

%************************************************************************************

4. The fitness of the members is recalculated using the function *fitness* and ranking them again into three groups according to fitness.

5. Variations are then applied to the shares (maintenance start times) of the members of the second and third group using the function *oscillation*. The code is also edited in order to cater for the integer maintenance start times.

6. Go back to step 2 and repeat until the program termination criterion is achieved.

The graph below shows the value of the best objective function as the iterations proceed.

Fig. 1.

**Fig. 1:**
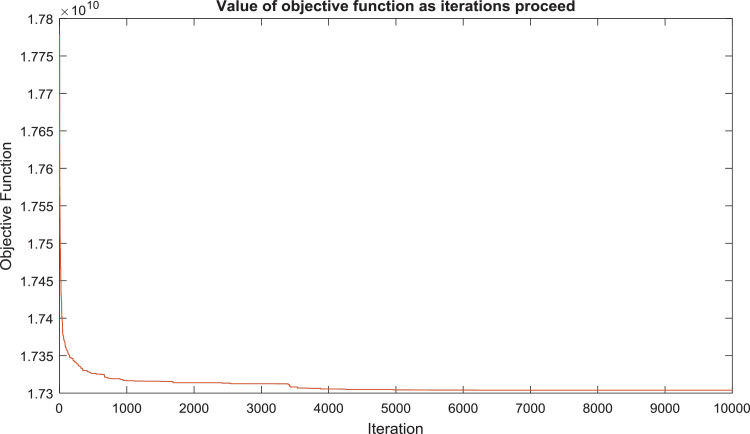
Value of objective function as iteration proceeds.

## Conclusions

This paper dealt with the application of Exchange Market Algorithm on the Generator Maintenance Scheduling problem. EMA has two search and two absorbent operators and hence it effectively seeks the global minima. The algorithm is successfully adapted to handle constraints and integer solutions.

Supplementary material *and/or* Additional information: The EMA code for the solving the generator maintenance schedule problem is attached.

## Declaration of Competing Interest

Authors declare no conflict of interest.
